# An Efficient Cell-Targeting Drug Delivery System Based on Aptamer-Modified Mesoporous Silica Nanoparticles

**DOI:** 10.1186/s11671-019-3208-3

**Published:** 2019-12-23

**Authors:** Yang Yang, Weihua Zhao, Wenwen Tan, Zongqiang Lai, Dong Fang, Lei Jiang, Chuantian Zuo, Nuo Yang, Yongrong Lai

**Affiliations:** 1 Department of Hematology, The First Affiliated Hospital of Guangxi Medical University, Nanning, 530021 Guangxi China; 2 Department of Pharmacy, The Second Affiliated Hospital of Guangxi Medical University, Nanning, 530007 Guangxi China; 3 Department of Surgery Oncology, Affiliated Tumor Hospital of Guangxi Medical University, Nanning, 530021 Guangxi China; 4 Department of Cardiothoracic Surgery, The First Affiliated Hospital of Guangxi Medical University, Nanning, 530021 Guangxi China

**Keywords:** Leukemia, Mesoporous silica, Drug delivery system, Sgc8, Targeted therapy

## Abstract

How to deliver chemotherapeutic drugs efficiently and selectively to tumor cells to improve therapeutic efficacy remains a difficult problem. We herein construct an efficient cell-targeting drug delivery system (Sgc8-MSN/Dox) based on aptamer-modified mesoporous silica nanoparticles that relies on the tumor-targeting ability of the aptamer Sgc8 to deliver doxorubicin (Dox) to leukemia cells in a targeted way, thereby improving therapeutic efficacy and reducing toxicity. In this work, Sgc8-MSN/Dox showed sustained Dox release, and they targeted and efficiently killed CCRF-CEM human acute T lymphocyte leukemia cells, suggesting potential as a cancer therapy.

## Introduction

Acute lymphoblastic leukemia (ALL) is a heterogeneous malignant tumor that seriously endangers human health [1–3]. About 6000 cases of acute lymphoblastic leukemia are diagnosed each year in the USA, especially in children and adolescents. ALL is the most common cancer among children and the most common cause of cancer death before the age of 20 [4].

Chemoradiotherapy and bone marrow hematopoietic stem cell transplantation are standard treatments for ALL. Radiotherapy is associated with substantial systemic side effects and the optimal sites of exposure are unclear. Bone marrow hematopoietic stem cell transplantation is often not feasible because of cost, patient age, and lack of donor marrow. Chemotherapy can be effective at eliminating distant foci and thereby prevent recurrence, but only a fraction of most anti-tumor drugs reach tumors via the circulation. This results in low drug bioavailability and poor selectivity for diseased cells over normal ones, increasing the risk of serious adverse reactions and drug resistance.

Nano-drug delivery systems can provide much more effective and targeted delivery of anti-tumor drugs than the drugs on their own, including drug release that is triggered by the specific conditions of the tumor microenvironment [5, 6]. Among such systems, mesoporous silica nanoparticles (MSNs) have attracted attention because of their large, modifiable surface; their adjustable pore volume, capable of carrying different amounts, and types of drugs; and their good biocompatibility [7, 8]. Surface modifications with functional response groups allow the creation of MSNs that release their drug under specific conditions, while modifications with targeting molecules lead the MSNs to deliver their drug selectively to desired tissue [9, 10].

One type of targeting molecule compatible with MSNs are aptamers, which are single-stranded DNA or RNA that act as “chemical antibodies” to bind specifically and tightly to targets. Aptamers are smaller and easier to prepare and modify than monoclonal antibodies, they penetrate tissue better and they show lower toxicity and immunogenicity. Therefore, aptamers are widely used for tumor detection and targeted chemotherapy [11–13]. The SELEX method has been used to identify an aptamer, Sgc8 that binds specifically to CEM human acute T lymphocyte leukemia cells [14, 15]. Sgc8 recognizes protein tyrosine kinase-7 (PTK-7) on the CEM cell membrane. Adding Sgc8 to chemotherapeutic drugs and certain nanomaterials can improve the targeting and killing of leukemia cells [16, 17].

**Fig. 1:**
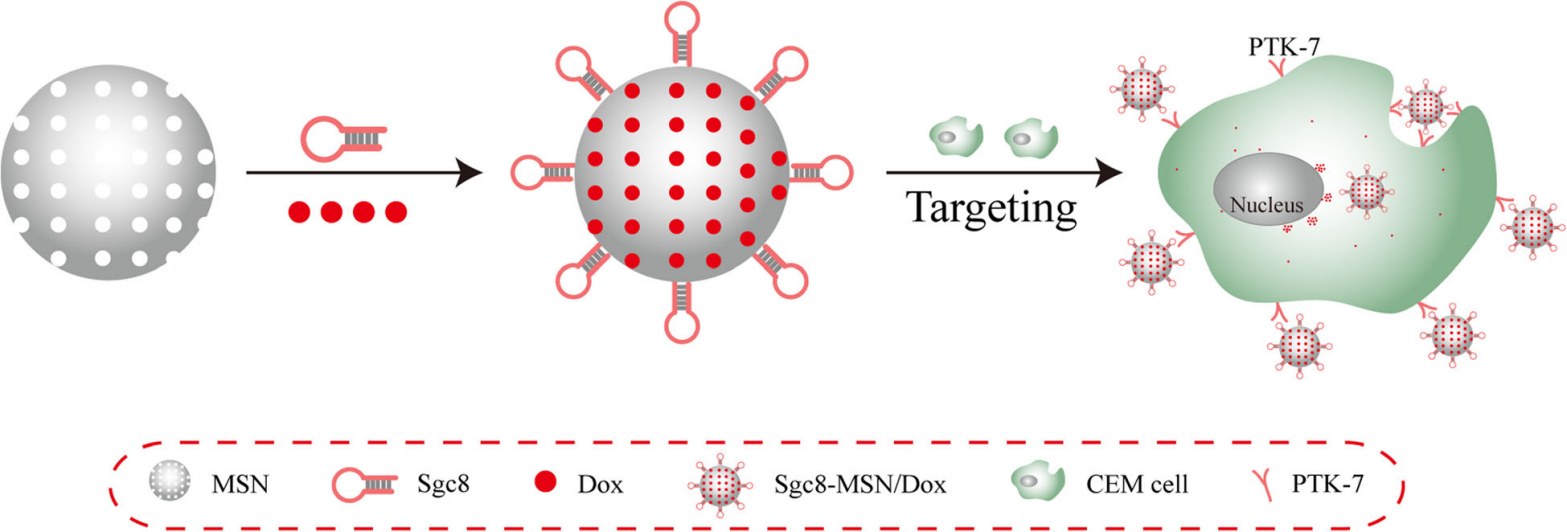
Schematic illustration of Sgc8 aptamer-modified mesoporous silica nanoparticles for an efficient cell-targeting drug delivery system. CEM cell, CCRF-CEM human acute T lymphocyte leukemia cells; Dox, doxorubicin; MSN, modified silica nanoparticle; PTK-7, protein tyrosine kinase-7

We have designed a Sgc8 aptamer-modified fluorescent silica nanoparticles system (Sgc8-FSNPs) for detecting leukemia cells with high sensitivity and specificity, which prove useful not only for the diagnosis of leukemia but also for the targeted delivery of anti-leukemia drugs [18]. Based on this work, in the present study, we encapsulated doxorubicin (Dox) in MSNs, which we decorated with Sgc8 (Fig. 1). The resulting Sgc8-MSN/Dox showed good morphology and size for drug delivery, and the nanoparticles were taken up selectively by leukemia cells in culture, leading to effective killing of tumor cells. The purpose of this study is to try to reveal that aptamer-modified meso porous silica nanoparticles can not only target the diagnosis of tumors, but also kill tumors by loading drugs, so as to provide ideas for tumor-targeting theranostics.

## Experimental Materials and Methods

### Reagents and Materials

Doxorubicin hydrochloride (Dox) was purchased from Beijing Huafeng United Technology (Beijing, China). Analytical-grade tetraethylorthosilicate (TEOS) and 3-Triethoxysilylpropylamine (APTES) were purchased from Shanghai Chemical Reagent Factory I (Shanghai, China), and cetyl-trimethylammonium bromide (CTAB, 99% purity) from Shanghai Jizhi Biochemical Technology (Shanghai, China). All other reagents were analytical-grade. Carboxyl-modified Sgc8 aptamer (5′-ATCTAACTGCCGCCGCGGGAAAATGTACGGTTAG(T)_10_-COOH-3′)[16]was synthesized by Shanghai Bioengineering (Shanghai, China).

### Cell Lines

The following cell lines were purchased from the Cell Bank of the Chinese Academy of Sciences (Shanghai, China): the human acute T lymphocyte leukemia cell line CCRF-CEM, the Ramos human Burkitt lymphoma B cell line, the L02 human normal liver cell line, and the 293Thuman embryonic kidney cell line. All cell lines were cultured at 37 °C under 5% CO_2_ in DMEM supplemented with 10% fetal bovine serum (FBS, Hyclone) and penicillin-streptomycin (Gibco, Grand Island, NY, USA).

### Preparation of Sgc8-MSN/Dox

MSNs were prepared as described [19] by dissolving 0.50 g CTAB in 240 mL of ultrapure water, adding 1.75 ml of 2 M NaOH solution, and heating at 80 °C in an oil bath. Under strong stirring, 2.50 mL TEOS was slowly added dropwise, and the mixture was stirred continuously for 2 h until a white precipitate was obtained. Then the mixture was centrifuged at 10,000 rpm for 10 min, the supernatant was removed, the precipitate was washed three times alternately with ultrapure water and anhydrous ethanol, and then the precipitate was dried in a vacuum oven at 60 °C overnight to obtain MSNs.

Sgc8-MSN/Dox was obtained by mixing 0.2365-g MSNs in a three-neck flask with 23.65 mL of absolute ethanol, adding 710-μL APTES dropwise, and refluxing for 24 h. The mixture was washed three times alternately with methanol-HCl solution (4:1) and deionized water, then dried to yield MSNs-NH_2_. At room temperature, MSNs-NH2 material was put in a 10-mL centrifuge tube and dispersed in a suitable amount of phosphate-buffered saline (PBS), then Dox (5 mg/mL) was added dropwise, and the mixture was stirred for 24 h. Finally, carboxyl-modified Sgc8 aptamer (100 nM/mL) was added, and the mixture was stirred for 1 h, centrifuged, and washed with PBS until it became colorless, yielding Sgc8-MSN/Dox.

### Characterization of Sgc8-MSN/Dox

X-ray diffraction patterns (XRD) of the nanoparticles were measured by a XRD D4 diffractometer (Bruker Inc., Germany). N2 adsorption−desorption isotherms were measured by specific surface area and pore size analyzer (TriStar 3000, GA Inc., USA). The BET surface areas were calculated from the BET plot. The pore size distribution was estimated from the adsorption branch of the isotherm by the BJH method. Particle size and electrostatic potential were measured using the Nano S dynamic light scattering analyzer (Malvern Instruments, UK). Measurements were taken three times for each sample and averaged. Particle size and morphology were also assessed by transmission electron microscopy (H-7650, Tokyo, Japan) at an electron beam acceleration voltage of 100 kV. Meanwhile, the conjugation of Sgc8 aptamer to the MSN surface was assessed by FT-IR spectroscopy (Nicolet-5700, USA). In order to detect the efficiency of aptamer-modified MSN/Dox, we collected the washing supernatant in the process of preparation Sgc8-MSN/Dox and measured the ultraviolet absorption value at 260 nm, and then using the standard curve method to determine the amount of unbound aptamer. So the amount of modification of aptamer can be calculated by subtracting the amount of unbound from the total amount added.

### Dox Release from Sgc8-MSN/Dox

Release of Dox from Sgc8-MSN/Dox was measured *in vitro* as follows. Sgc8-MSN/Dox (10 mg) was dissolved in 10 mL of buffer at pH 5.0 or 7.4 at 37 °C and shaken at 100 rpm. At predetermined times, samples were removed, centrifuged at 5000 rpm for 10 min, and the supernatant was assayed for Dox using spectrophotometry as described by Thermo Scientific Microplate Reader (81 Wyman Street, Waltham, USA) at 482 nm [9].

### Targeting Ability of Sgc8-MSN/Dox

CEM or Ramos cells (3 × 10^6^) in PBS were added to 15-mL centrifuge tubes and mixed with Sgc8-MSN/Dox or MSN/Dox (aptamer concentration, 200 nM). Tubes were incubated at 37 °C for 20–30 min, and then cells were collected by centrifugation, washed 3 times with PBS, fixed with 4% paraformaldehyde for 30 min, and washed 3 times with PBS. Cells were incubated for 5 min with 1 mg/ml DAPI, washed 3 times with PBS and centrifuged. The cell pellet was resuspended in PBS and placed onto slides for analysis by fluorescence confocal microscopy ((Nikon DS-Ri1; Tokyo, Japan).

In separate experiments, CEM and Ramos cells (3 × 10^5^) in logarithmic growth phase were resuspended in a mixture of 50 μL of PBS, 45 μL of binding buffer [PBS supplemented with 5 mM MgCl2, 4.5 g/L glucose and 1 mg/ml bovine serum albumin (BSA)], and 10 μL of FBS [PBS supplemented with 1 mg/ml bovine serum albumin (BSA)] containing the indicated materials at an aptamer concentration of 200 nM. The mixture was shaken in the dark for 30 min at 4 °C. Cells were washed in wash buffer, centrifuged at 1000 rpm for 5 min, then washed another 3 times. Finally, cells were resuspended in 500 μL of wash buffer and analyzed by flow cytometry (Beckman Coulter Epics X L; Beckman Coulter, Inc., Brea, CA, USA).

### Cytotoxicity of MSNs

Cytotoxicity of MSNs without Dox or aptamer was assessed using the MTT method. CEM, Ramos, 293T, and L-02 cells in logarithmic growth phase were added to 96-well plates (1.5 × 10^4^/well). MSNs (100 μL) were added to each well, and the plates were cultured in a 5% CO_2_ incubator at 37 °C for 24 or 48 h. MTT (20 μL) was added to each well, the plates were incubated at 37 °C for another 30 min, then the plates were centrifuged at 1500 rpm for 10 min, and culture supernatants were discarded. DMSO (200 μL) was added to each well, the plates were shaken in the dark for 10 min, and then optical density (OD) at 570 nm was measured using an automated microplate reader. Each sample was tested with six replicates, and results were expressed as mean ± SD.

### Cell killing by Sgc8-MSN/Dox

CEM, Ramos, 293T, and L-02 cells in 96-well plates (1 × 10^5^ cells per well) were incubated for 24 h with free Dox, MSN/Dox, or Sgc8-MSN/Dox at Dox concentrations of 1, 5, 10, 15, or 20 μg/mL. In order to further confirm that Sgc8-MSN/Dox was indeed targeted to kill CEM cells through aptamer receptor, we first incubate sufficient Sgc8 aptamer with CEM cells for 2 h, then incubated with sgc8-MSN/Dox at Dox concentration of 20 μg/ml for 24 h. Viability was compared using the MTT assay as described in the “Cytotoxicity of MSNs” section.

### Statistical Analysis

Results were analyzed statistically using Student’s *t* test and one-way analysis of variance with the least significance difference test. All analyses were performed with GraphPad Prism 6.02 (GraphPad Software, San Diego, CA, USA).

## Results and Discussion

### Characterization of Sgc8-MSN/Dox

**Fig. 2:**
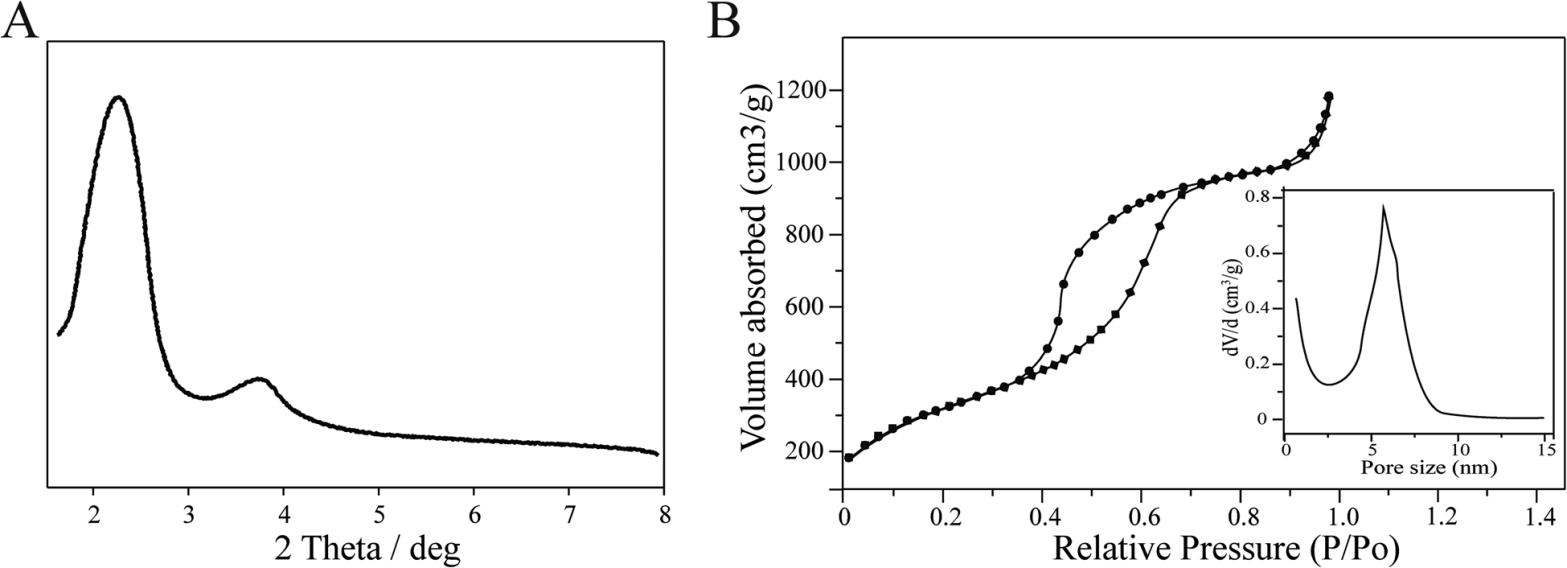
**a** XRD and **b** nitrogen sorption isotherms of MSN; (inset) Pore volume and pore size distribution plots of MSN

**Fig. 3:**
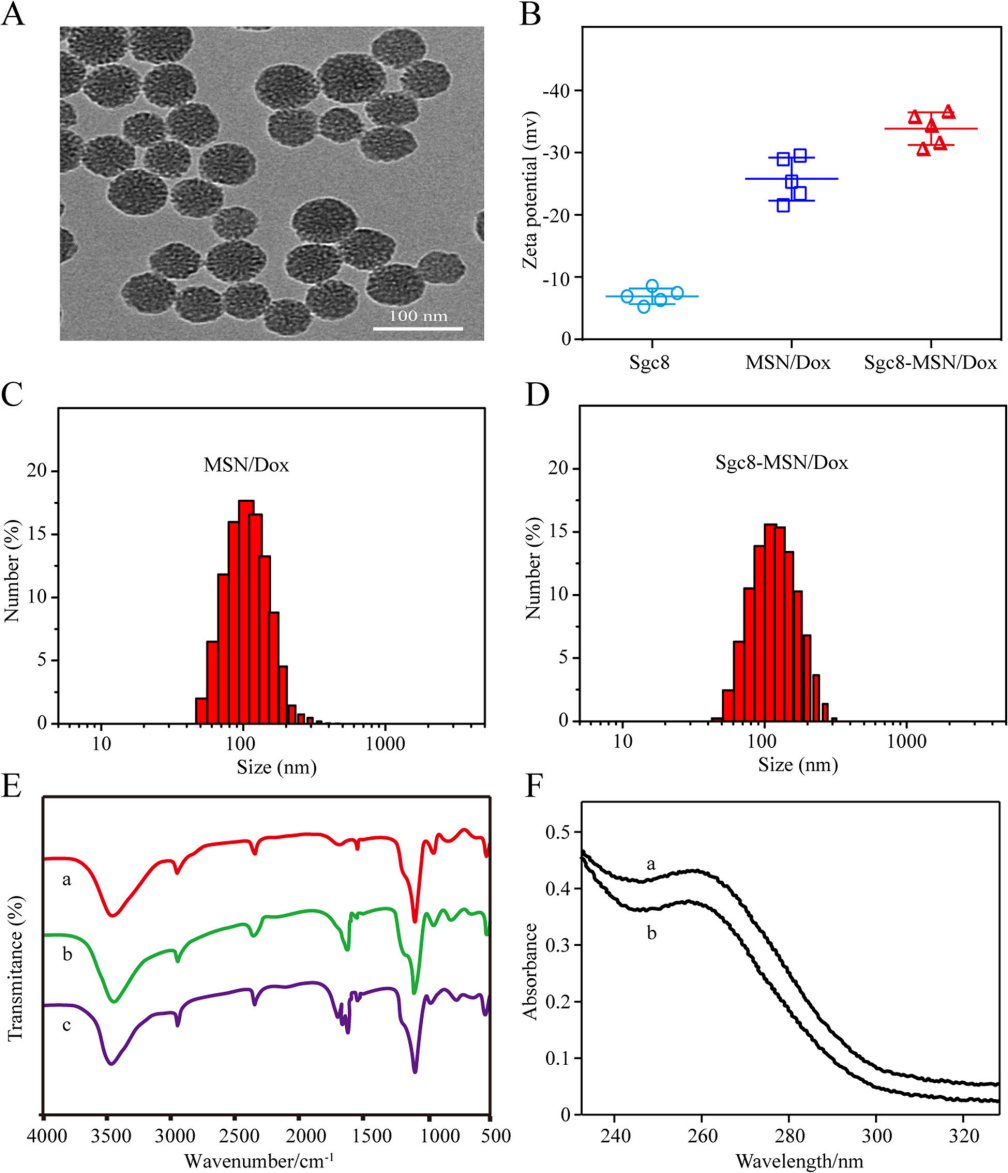
Characterization of nanomaterials. **a** Electron micrograph of Sgc8-MSN/Dox. **b** Potential diagram of nanoparticles. Particle size distribution of **c** MSN/Dox and **d** Sgc8-MSN/Dox. **e** FT-IR spectra of nanomaterials: **a** MSN, **b** MSN/Dox, and **c** Sgc8-MSN/Dox. **f** UV–Vis spectra of **a** Sgc8 aptamer solution, **b** washing supernatant in the process of preparation Sgc8-MSN/Dox

X-ray diffraction data show that the synthesized nanoparticles have a typical diffraction peak of hexagonal mesopores in the X range (Fig. 2a), which confirmed the structure of MSN. In order to further study the specific surface area, pore size distribution, and mesoporous parameters of MSN, we performed a nitrogen adsorption–desorption test on MSN. The N2 adsorption−desorption isotherms of MSN (Fig. 2b) exhibit a type IV isotherm characteristic, indicating mesoporous characteristics. The surface area calculated by BET model is 1389 m^2^/g. BJH curve shows that the pore size distribution of the particles is narrow (Fig. 2b, inset). The pore size is 5.23 nm, and the pore volume is 2.51 cm^3^/g. The large specific surface area and pore volume and rich mesopores make it possible to have a stronger drug loading capacity and have the potential to be an excellent drug carrier. Electron microscopy showed Sgc8-MSN/Dox to have good dispersibility and uniform size, with spherical or elliptical shape (Fig. 3a). The average zeta potential of MSN/Dox in PBS was − 26.53 mV, which decreased to − 33.87 mV in Sgc8-MSN/Dox (Fig. 3b), reflecting the negative charge on the Sgc8 aptamer [20]. Pore channels on the surface of the mesoporous silicon were observed. In PBS, MSN/Dox showed an average hydration particle size of 98.35 nm, which increased to 103.24 nm after linking the Sgc8 aptamer to make Sgc8-MSN/Dox (Fig. 3 c, d). To further confirm the successful conjugation of Sgc8-MSN/Dox, FT-IR spectroscopy analysis was performed. The infrared spectrum is shown in Fig. 3 e. Peaks of 3400 cm^−1^, 1100 cm^−1^, and 500 cm^−1^ are characteristic peaks of silica. The peak about 2400 cm^−1^ is the peak of CTAB template agent. The peak about 1600 cm^−1^ is NH_2_ peak. The peak appearing around 1700 cm^−1^ is considered as the C = 0 bond stretching characteristic peak of cluster base of Dox loaded in MSNs. The new peak at 1650 cm^−1^ is due to the Schiff base (–C=N–) generated by the reaction of Sgc8 with NH2-MSN/Dox. These evidence proves that Sgc8-MSN/Dox drug delivery system has been successfully prepared. In order to detect the efficiency of aptamer-modified Sgc8-MSN/Dox, we used an ultraviolet spectrophotometer to measure the absorbance density (OD) of aptamer at 260 nm, and calculated the aptamer modification to nanoparticle efficiency based on the change in OD value before and after aptamer modification to the surface of the nanoparticle. The ultraviolet absorption (UV–Vis) spectra of aptamer solution and supernatant are shown in Fig. 3e. According to the calculation, the amount of Sgc8 aptamer modification to Sgc8-MSN/Dox was about 6.87 nmol mg^–1^ Sgc8-MSN/Dox.

### Dox Release from Sgc8-MSN/Dox In Vitro

**Fig. 4:**
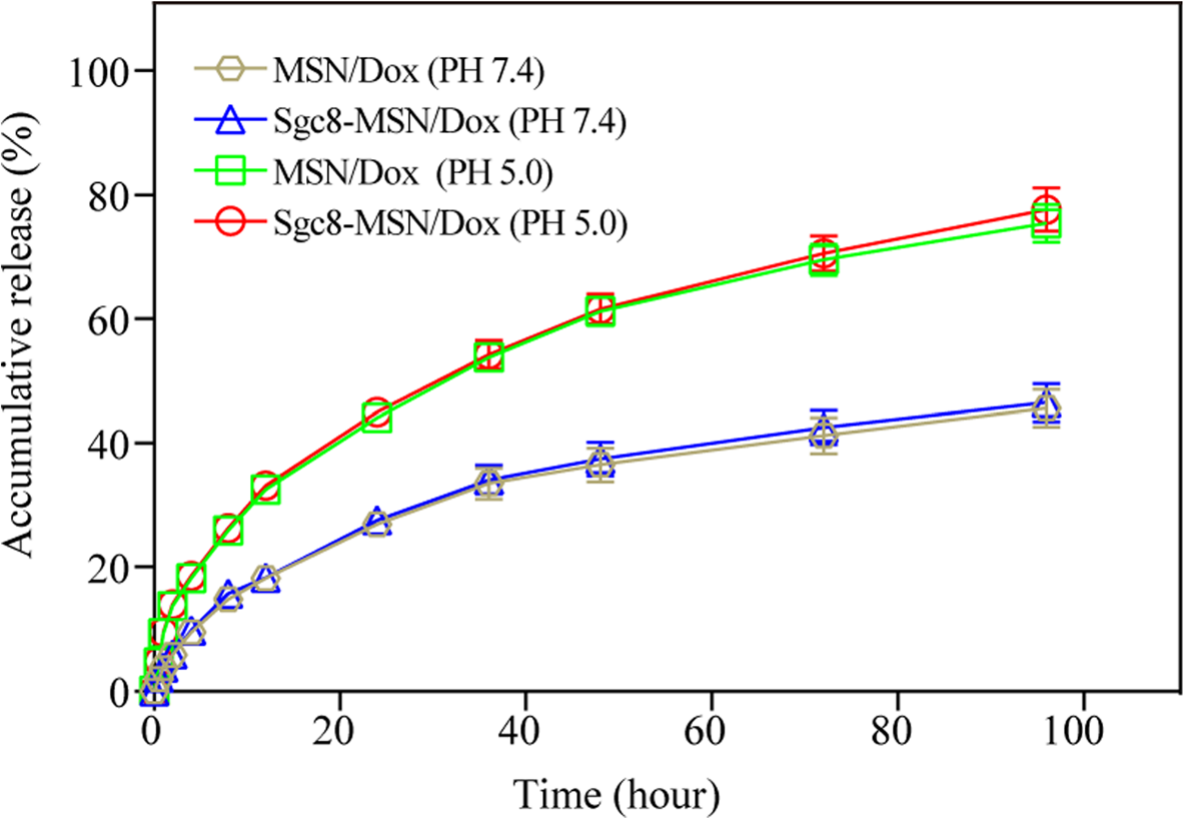
Release of doxorubicin from Sgc8-MSN/Dox and MSN/Dox in vitro at pH 5.0 or 7.4

MSN/Dox and Sgc8-MSN/Dox released their drug cargo slowly at pH 7.4: no more than 50% of the drug was released within 96 h (Fig. 4). This indicates that MSNs can support slowly drug release lasting 96 h, likely because the drug spreads throughout the internal channels of the mesoporous silicon. Release was much faster at pH 5.0, with release rates reaching 60% by 48 h and > 80% by 96 h. Dox release curves were nearly identical for Sgc8-MSN/Dox and MSN/Dox, suggesting that aptamer ligation does not affect the nanoparticle structure. The greater release at acidic pH is useful because tumor cells are in a weakly acidic environment and Sgc8-coated nanoparticles are internalized into endosomes [15]. Our results support the idea that mesoporous silicon can accelerate drug release in acidic environment [21, 22].

### Targeting of Leukemia Cells by Sgc8-MSN/Dox In Vitro

**Fig. 5:**
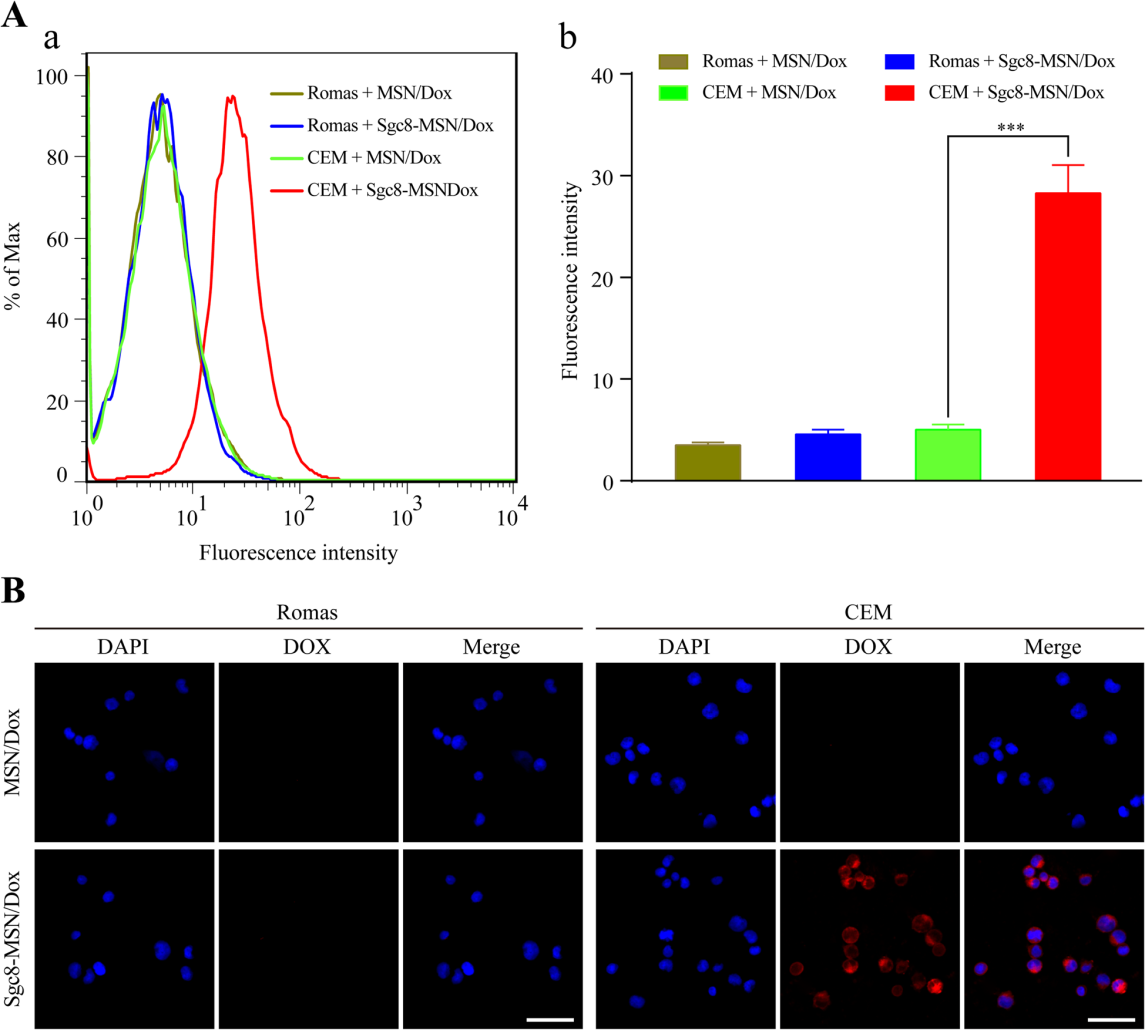
In vitro targeting of leukemia cells by Sgc8-MSN/Dox analyzed using **a** flow cytometry and **b** fluorescence microscopy. CCRF-CEM and Romas cells were incubated with MSN/Dox orSgc8-MSN/Dox, and then stained with DAPI (blue). Dox appeared red. Scale bar, 100 μm

We examined nanoparticle uptake by CEM T lymphocyte leukemia cells as target cells and Ramos B lymphoma cells as non-target cells. Based on flow cytometry, non-target cells internalized MSN/Dox to a similar extent as Sgc8-MSN/Dox, whereas target cells internalized the aptamer-coated nanoparticles to a much greater extent than MSN/Dox (Fig. 5a). Consistent with these results, fluorescence confocal microscopy showed strong Dox fluorescence (red) within CEM cells exposed to Sgc8-MSN/Dox, but not within Ramos cells treated in the same way (Fig. 5b). These results reflect the established ability of Sgc8 aptamer to target leukemia cells [20]. Sgc8 recognizes PTK-7 on the surface of CEM cells [18], recruiting nanoparticles to the surface and thereby making their uptake more efficient [23]. Our results suggest that Sgc8-MSN/Dox may target PTK-7-expressing cancer cells at primary as well as secondary sites or in circulation, making it useful for controlling metastasis. It may be possible to expand our MSN approach to other aptamers with other targeting specificities [24, 25].

### Cytotoxicity of MSNs

**Fig. 6:**
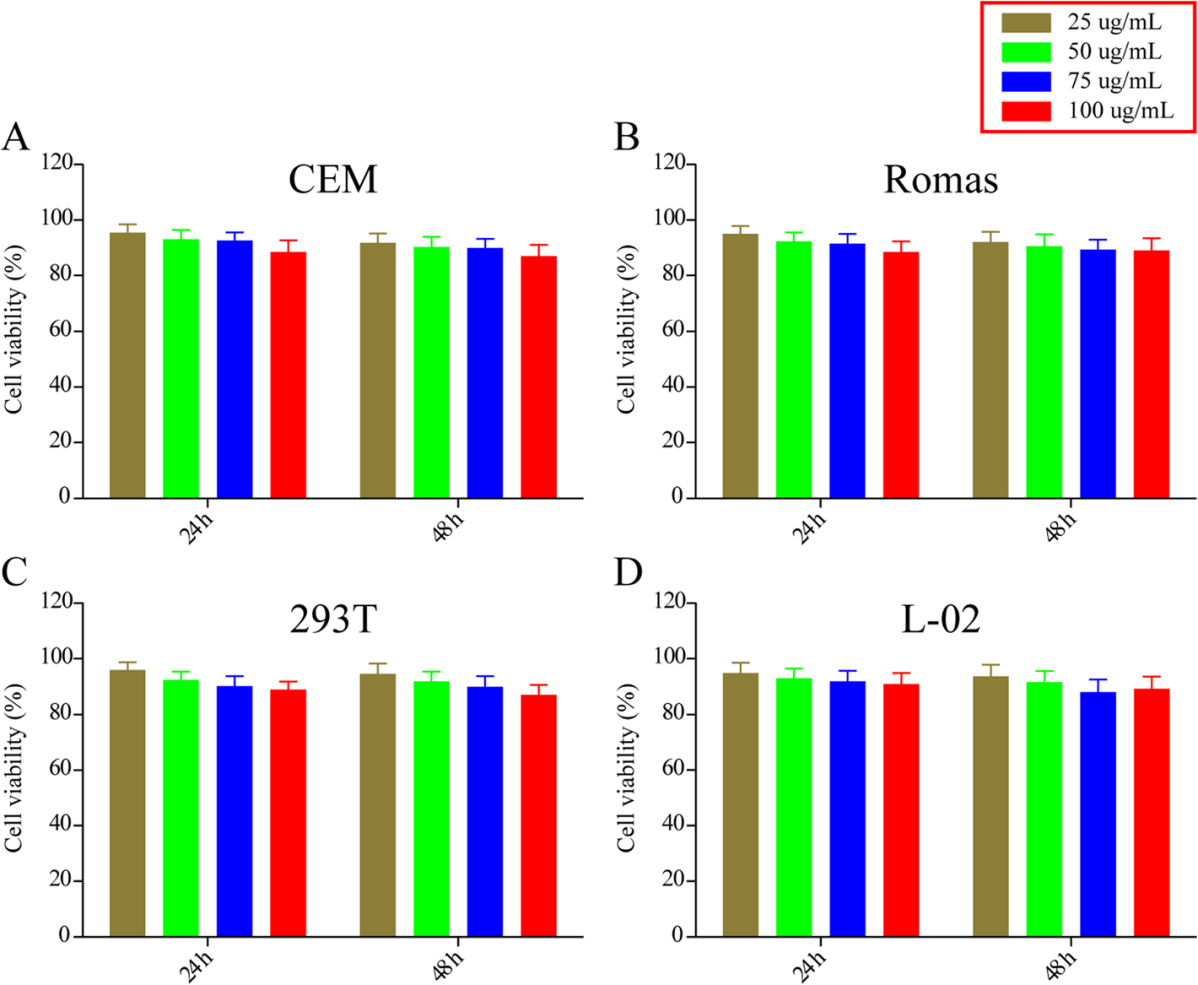
In vitro cytotoxicity of MSNs. **a** CEM, **b** Ramos, **c** 293T, and **d** L-02 cells were treated with the indicated concentrations of MSN, and cell viability was measured

Given the importance of biosafety for nano-drug delivery systems [25], we first examined the toxicity of blank MSNs against CEM, Ramos, 293T, and L-02 cells. Viability of all cell lines remained above 90% even after 48-h incubation with up to 100 μg/mL MSNs (Fig. 6). This suggests that MSNs show nearly negligible cytotoxicity.

**Fig. 7:**
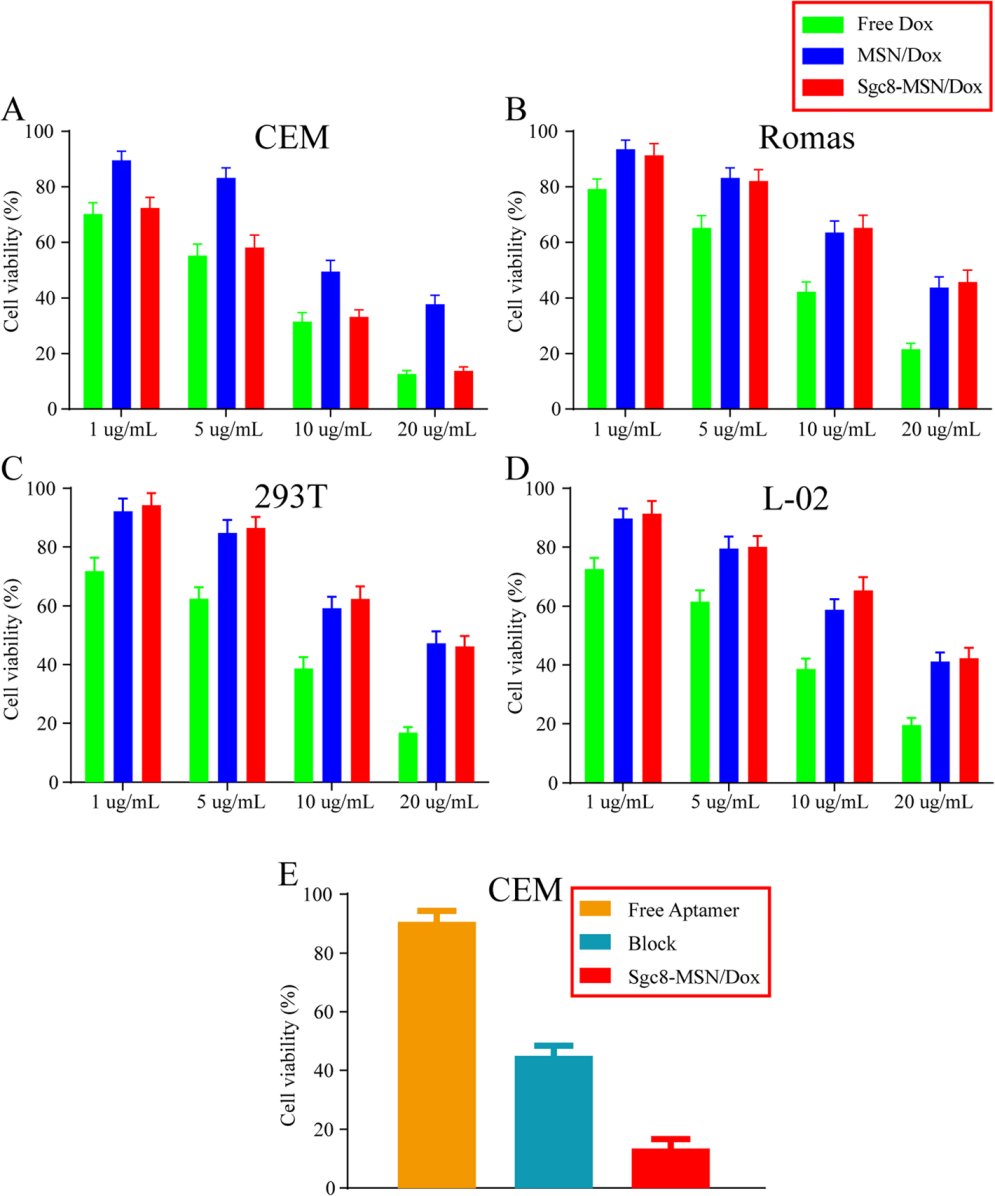
Ability of free DOX, MSN/Dox, and Sgc8-MSN/Dox to kill **a** CEM cells, **b** Ramos cells, **c** 293T cells, and **d** L-02 cells. **e** CEM cells killing ability of free aptamer, block (CEM cells first incubate sufficient Sgc8 for 2 h, then incubated with sgc8-MSN/Dox), and Sgc8-MSN/Dox

Next, we investigated the ability of Sgc8-MSN/Dox to kill target tumor cells (CEM cells) and non-target cells (Ramos, 293T, and L-02 cells). The cells were incubated with different concentrations of free Dox, MSN/Dox, or Sgc8-MSN/Dox for 24 h, and then viability was assessed using the MTT assay. Against target CEM cells, free Dox showed slightly greater toxicity than the two MSN formulations, while Sgc8-MSN/Dox was significantly more toxic than MSN/Dox at the same drug concentration (Fig. 7). Against Ramos, 293T, and L-02 cells, free Dox showed significantly greater toxicity than the MSN formulations, which showed similar toxicity with or without Sgc8. In order to further confirm that Sgc8-MSN/Dox was indeed targeted to kill CEM cells through aptamer receptor PTK-7, we first incubate sufficient Sgc8 aptamer with CEM cells for 2 h to block the binding site and then co-incubated with the Sgc8-MSN/Dox. The MTT assay showed that free aptamer had no toxicity to CEM cells, and after sufficient Sgc8 aptamer blocked the binding site, Sgc8-MSN/Dox showed significantly less toxicity than free Sgc8-MSN/Dox group (Fig. 7e). These results highlight the ability of the aptamer to target drug delivery and enhance the killing of target cells. Such targeting can help reduce the uptake of drug by non-target cells as well as permit the use of lower doses; both effects can reduce the risk of toxic side effects [26].

Aptamers can be used as guiding molecules for the delivery of drugs and various macromolecules or nanocarriers to tumor cells, sometimes aptamer also can work as therapeutic. For example, AS1411 aptamer can bind to nucleolin specifically, and nucleolin is expressed in and on the surface of tumor cells involved in cell differentiation, survival, inflammation, angiogenesis, and tumor development. AS1411 aptamer can induce growth inhibition of xenograft models (renal cancer, lung cancer, MX1 breast cancer, and pancreatic cancer) by binding with nucleolin [27]. Moreover, AS1411 aptamer-functionalized, doxorubicin-loaded liposomes (Apt-Dox-Lip) were tested on breast cancer, and the results showed great application potential [28]. Aptamers have been successful in a wide range of clinical trials owing to their excellent properties. The first aptamer was approved by the FDA in 2005 [29], since then, more and more aptamers have reached clinical trials. Aptamers were applied to anti-tumor clinical trials, including prostate cancer [30], lung cancer [31], acute myeloid leukemia [32], and so forth. It is no doubt that these aptamer-related clinical trials give a new hope to change the conventional style of therapy.

In recent years, aptamers have been combined with different nanomaterials for targeted therapy of tumors. It can be seen that aptamers have extraordinary molecular recognition and targeting capabilities. However, there are still some problems that cannot be ignored in the development of application of aptamers, such as whether nuclease affects the stability of aptamers in vivo, whether the aptamers of small molecular substances enter the body and are easy to be quickly removed by the renal system, and whether the actual targeting performance in vivo is feasible. In order to exploit the full potential of aptamer-based targeted nanomaterials, more in vivo testing and clinically relevant experiments remain the focus of current research. At the same time, theranostics of tumors is an important direction for future development, and the design and preparation of more multifunctional biological functional materials are undoubtedly the development trend.

## Conclusion

We have constructed an aptamer-modified MSN delivery system that can efficiently target leukemia cells and promote Dox uptake. This targeting, coupled with the ability of MSNs to release drug cargo slowly and preferentially under acidic conditions, may help the delivery system accumulate at tumor sites and kill the cells continuously. It may be possible to target nearly any type of tumor cells with this MSN approach by selecting suitable aptamers. But it cannot be ignored that the Sgc8-MSN/Dox may also have the problems mentioned above, and its in vivo stability and targeting still need further study. In the next, we will combine the previous research to load the drug delivery system with both diagnostic reagents and drugs, giving it the theranostic function of targeted diagnosis and targeted treatment.

## Abbreviations

MSN: Mesoporous silicon nanoparticles
Dox: Doxorubicin
Sgc8-MSN/Dox: Aptamer-modified mesoporous silica drug delivery system
PTK-7: Protein tyrosine kinase-7
TEOS: Tetraethylorthosilicate
APTES: 3-Triethoxysilylpropylamine
CTAB: Cetyl-trimethylammonium bromide
CEM: CCRF-CEMcells
PBS: Phosphate-buffered saline
